# A retrospective analysis of elective lower gastrointestinal endoscopy in patients 80 years of age and older

**DOI:** 10.1186/1471-2482-13-S1-A12

**Published:** 2013-09-16

**Authors:** Giovanni D De Palma, Bruno Amato, Saverio Siciliano, Francesco Maione, Dario Esposito, Nicola Gennarelli, Marcello Persico, Stefania Masone, Giuseppe Iannone, Pietro Forestieri

**Affiliations:** 1 Department of Gastroenterology, Endocrinology and Surgery. Center of Excellence for Technical Innovation in Surgery, University of Naples Federico II, School of Medicine, Italy

## Introduction

Colonoscopy is the standard investigation for colonic disease. However, clinicians often are reluctant to refer elderly for the procedure because of a perception of a greater risk of complications and high failure rates.

Our aims are to assess the success rate, safety and complications associated with colonoscopy performed in patients 80 years of age and older.

## Methods

Patients aged 80 years or over referred for colonoscopy from January 2010 to December 2012, were identified and retrospectively reviewed. Follow-up examinations, for previous colonic resection or polypectomy, assessment of IBD, and urgent colonoscopy for acute hemorrhage, were excluded for evaluation.

The patients were prepared for colonoscopy by ingesting 4 L of polyethylene glycol (PEG) a day before the procedure. The use and dosage of sedative drugs and/or antispasmodic agents administered was at the discretion of the endoscopist.

The primary aims were the complete examination, the main diagnosis and complication. A complete examination was defined as visualization of the ileocecal valve and/or ileal intubation.

## Results

One hundred and twelve patients (61 females, median age 84 years, rage 80-97 years) were included in the study. The main indications were anemia (63,4%), diarrhea (19,6%), abdominal pain (8,9%), and altered bowel habit (8,1%).

A complete examination was achieved in 96 patients (85.7%). Reasons for failure to reach cecum were poor bowel preparation (11 patients, 9.8%), impassable stricture (3 patients, 2,7%), major hypotension (1 patient, 0,9% ), and technical difficulty (1 patient, 0,9%).

The main diagnosis included adenoma in 14 patients (12.5%), cancer in 9 patients (7.9%), vascular lesion in 9 patients (7.9%) drugs associated colitis in 6 patients (5.3%) and diverticular stenosis in 4 patients (7.1%).

Minor events occurred in 10 patients (8.9%), (desaturation 7, transient hypotension 3).

## Conclusion

Our study showed that colonoscopy in patients 80 or more years of age is safe, effective, and has a high diagnostic yield. The procedure can be completed in over 85%. The complication are usually transient and related to sedation. Colonoscopy should be the preferred method of colonic investigation in elderly patients.
